# The generality of the critical spacing for crowded optotypes: From Bouma to the 21st century

**DOI:** 10.1167/jov.21.11.18

**Published:** 2021-10-25

**Authors:** Daniel R. Coates, Charles J. H. Ludowici, Susana T. L. Chung

**Affiliations:** 1 College of Optometry, University of Houston, Houston, TX, USA; 2 School of Optometry, University of California, Berkeley, Berkeley, CA, USA; 3 School of Optometry, Vision Science Graduate Group, University of California, Berkeley, Berkeley, CA, USA

## Abstract

It is rare to find a crowding manuscript that fails to mention “Bouma's law,” the rule of thumb stating that flankers within a distance of about one half of the target eccentricity will induce crowding. Here we investigate the generality of this rule (even for just optotypes), the factors that modulate the critical spacing, and the evidence for the rule in Bouma's own data. We explore these questions by reanalyzing a variety of studies from the literature, running several new control experiments, and by utilizing a model that unifies flanked identification measurements between psychophysical paradigms. Specifically, with minimal assumptions (equivalent psychometric slopes across conditions, for example), crowded acuity can be predicted for arbitrary target sizes and flanker spacings, revealing a performance “landscape” that delineates the critical spacing. Last, we present a compact quantitative summary of the effects of different types of stimulus manipulations on optotype crowding.

## Introduction

Crowding, the interference of the recognition of an object due to the presence of proximal objects, has garnered a lot of interest in the vision science community over the past two decades. The appeal of the phenomenon to the vision research community is likely because crowding is one of the very few phenomena that resonates with a variety of disciplines, ranging from clinical optometry/ophthalmology, learning disability, psychology, cognitive science, neuroscience, and computational modeling. The interest in the phenomenon differs across disciplines. For instance, clinical vision scientists are interested in crowding because of its relevance to acuity testing and its potential to help diagnose visual disorders (Flom, Heath, et al., 1963, Flom, Weymouth, et al., 1963). To our knowledge, most of the earliest investigations on crowding came from clinical observations of people with amblyopia having difficulty reading letter charts when the letters were close together. Ehlers (1953) first described how the region over which letters could be recognized “narrows” when “the visual field is crowded with letters.” Stuart and Burian (1962) coined the term *separation difficulty* to describe the phenomenon. Educators are interested in crowding because of the common observation or complaint that people with dyslexia often make letter reversal errors when reading, which is commonly believed to be the contributing factor of the reading deficits of people with dyslexia (Bouma & Legein, 1977; Geiger & Lettvin, 1987; Atkinson, 1991), although the issue is still not yet resolved (Martelli et al., 2009; Shovman & Ahissar, 2006). Psychologists, cognitive scientists, and neuroscientists are interested in crowding because crowding is suggested to be the bottleneck on object recognition (Levi, 2008; Pelli & Tillman, 2008), and thus understanding crowding, especially the cortical site of where crowding occurs, might help us uncork the bottleneck on the recognition of other objects in general. The richness of empirical data on the phenomenon also provides a fertile ground for computational scientists to formulate and validate their models.

Undoubtedly, the multidisciplinary interest in the crowding phenomenon has substantially improved our knowledge of the phenomenon while, at the same time, provides a rich medium of data. Unfortunately, it is often difficult to take advantage of the rich data available in the literature because studies from different disciplines often use different methods to collect data—including tasks, psychophysical methods, variables to be measured, target size, number of flankers, type of flankers, and so on. Not only do these differences make it challenging to compare a measurement across studies, but also they could lead to *seemingly* contradictory conclusions. For example, in clinical settings, performance for reading letters (or other optotypes) on a letter chart, with or without nearby objects such as other letters or bars (i.e., flankers), is often measured for different letter sizes. When flankers are present, both the target and flankers would vary in size together to maintain a fixed relative separation between them (the *nominal* spacing). This paradigm implies crowding might depend on target size. Indeed, the smallest letters that can be read (acuity) are often smaller when letters are presented alone, compared with when flankers are present. In contrast, in laboratory settings, the predominant paradigm used to study crowding is the measurement of performance for recognizing a target of a suitable fixed size, while varying the target-flanker spacings. Studies using this paradigm have found that crowding is mostly *independent* of the target size. Which view is correct? Do these two paradigms really lead to different conclusions? Here, we show how to reconcile these seemingly contradictory observations and demonstrate that these two methods of measuring crowding are fully relatable and that they simply sample the space of crowded performance in different ways.

Crowding can be quantified in terms of its *magnitude* (the decrement in performance of recognizing the target in the presence of flankers) and *extent* (the spatial spread of the effect). In this article, most of the discussion will be focused on the extent, more commonly referred to as the *critical spacing*, rather than magnitude, simply because most crowding studies used the critical spacing as their measurement parameter. The popularity of studying critical spacing rather than the magnitude of crowding is likely because critical spacing scales with eccentricity—a relationship that constitutes one of the diagnostic criteria for crowding (Whitney & Levi, 2011; Pelli et al., 2004) and may reflect the retinotopy of the visual cortex as well as changes in receptive field sizes with eccentricity (Levi, 2008; Pelli, 2008). Considering that the measurement of critical spacing is sensitive to many stimulus or experimental factors, here, we asked the question of whether there are some general and universal relationships that govern the effect of certain stimulus or experimental factors on critical spacing. We identified several factors that we believe are fundamental to any psychophysical studies on crowding, which, depending on the choice of parameters, could have a significant impact on critical spacing. Experimental factors include the subject's task, psychometric function fitting, and criteria for defining threshold and measurement parameters. Stimulus factors include target size, contrast, and duration. To gain a comprehensive understanding of how each of these factors affects critical spacing, we reviewed relevant studies in the literature, reanalyzed data of some published studies, and collected empirical data to fill in the knowledge gap on some stimulus factors. A general model of crowded letter acuity is introduced to extrapolate arbitrary psychometric functions across eccentricities, letter sizes, and contrasts.

We will restrict our discussion to one general type of stimulus, namely, optotypes, to search for general principles governing critical spacing. Previous studies have shown that critical spacings are similar when crowding is measured using letters or everyday objects (Wallace & Tjan, 2011) or when measured between parts of a word (i.e., letters) or of a face (e.g., mouth, nose, eyes; Martelli et al., 2005). Therefore, we believe that any general principles that we learn from this study are likely applicable to other types of stimulus. The choice of optotypes/letters over other stimuli is primarily because letters are everyday objects and are highly learned, so that the inability to recognize them could not be attributed to an unfamiliarity effect. Simpler stimuli such as lines or patches of sine-wave gratings, although also popular in crowding studies, especially those dealing with computational modeling, sometimes could lead to confounding effects such as a release of the target from crowding when the flankers become more salient (increase in number or size of flankers)—a grouping effect (Levi & Carney, 2009; Livne & Sagi, 2010; Manassi et al., 2012; Saarela et al., 2009). In addition, the crowding effect obtained using lines or sine-wave gratings is strongly susceptible to the configurational and contextual effects of target and flankers (Livne & Sagi, 2007, 2010, 2011; Manassi et al., 2012; Saarela et al., 2009).

A signature of crowding is that the critical spacing scales with eccentricity. Pelli et al. (2004) suggested that this can be used as a diagnostic test for crowding. Mathematically, the critical spacing can be expressed as a proportion of the eccentricity, for eccentricities above approximately 1 degree of eccentricity (Strasburger, 2020). The proportionality constant, which we call the Bouma fraction (note that Bouma never used this term), is widely cited as 0.4 to 0.5 (Bouma, 1970; Pelli et al., 2004), although lower values (∼0.3) have also been reported (Chung et al., 2001; Kooi et al., 1994; Toet & Levi, 1992; Strasburger et al., 1991), and values as low as 0.1 to 0.2 have been reported for the tangential dimension (Chung et al., 2001; Levi, 2008). A Bouma fraction of approximately 0.4 is the critical spacing that corresponds to a constant length of approximately 6 mm of cortex in V1 (Pelli & Tillman, 2008), although Strasburger (2020) has shown that the relation only holds for eccentricities above 5 to 10 degrees. This distance matches the length of horizontal connections in V1 (Gilbert & Wiesel, 1989; Gilbert & Li, 2012), suggesting that these horizontal connections might mediate the lateral interactions observed in crowding. Note, however, that critical spacing has been shown to become smaller with practice (Chung, 2007; Chung et al., 2012; Chung & Truong, 2013) the “uncrowding” effect, which may be explained by a change mediated by the horizontal connections (Gilbert & Li, 2012). In any case, to facilitate the comparison of critical spacing from different studies that tested at various eccentricities, we will compare the Bouma fraction (ratio of critical spacing to eccentricity) instead of the physical size of critical spacing in several sections in this article.

This article has several goals concerning the generality of the critical spacing for crowding, including the measurement of the functions that reveal it. First, we reanalyze the empirical data reported by Bouma (1970) to illustrate the issues concerning the experimental determination of crowding functions, particularly with respect to practical and theoretical concerns. To assist in this process, we introduce a model of flanked acuity that can be used to extrapolate arbitrary psychometric functions for crowding. Then, we show how stimulus parameters affect crowding, particularly contrast and duration. By incorporating a large set of data from previous literature, we show how results can be reconciled and compared across studies that used seemingly different experimental paradigms.

## The crowding function

### Bouma (1970)

**Figure 1.**
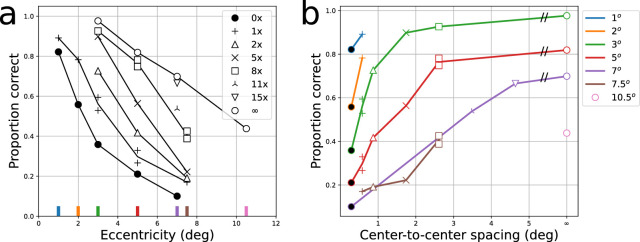
Replotting of Bouma (1970). Panel on left mimics the style used by Bouma, with eccentricity plotted on the abscissa, and distinct curves for each nominal edge-to-edge target-flanker spacing (number of 0.29 degree spaces between each letter; “0×” indicates abutting flankers). In the right panel, the same data are replotted with center-to-center absolute target-flanker spacing on the abscissa (with unflanked shown as the right most symbols, at “infinity”), with the curves for different eccentricities shown in different colors. The same symbol types, indicating nominal flanker spacing, are used for the both panels. Small colored lines on left plot indicate corresponding eccentricity color on right plot. Abscissa in left plot indicates edge-to-edge spacing (following Bouma), while right-side abscissa shows center-to-center spacing (edge-to-edge spacing +0.29 degrees).

All crowding researchers are intimately familiar with the “rule of thumb” laid out in the seminal article from Bouma (1970): The critical spacing for crowding is approximately half of the eccentricity, and numerous researchers have refined and augmented this formulation (Rosen et al., 2014; Strasburger, 2020). Regardless of its validity, here we step through the evidence for the “rule” in the original study itself. First, to review the experiment details, Bouma presented lowercase Courier letters with an x-height of approximately 14 min of arc at multiple locations in the visual field, presented randomly to the left or right of fixation for 200 ms. Letters were presented alone or flanked on both sides by the letter x at different spacings (multiples of empty letter slots between a pair of adjacent letters, with each letter slot equivalent to 0.29 degrees). He measured the ability of subjects to correctly identify the central letter in these conditions and presented curves like those shown in Figure 1a, which we replotted from his data. He stated that for correct identification, “no other letter should be present within 0.5 times [the eccentricity of the target letter].” How did he derive this rule from the data shown in Figure 1a? Consider performance at 5 degree eccentricity. When unflanked, the ability to identify an isolated letter (top-most curve, open circles) was approximately 80%. At 8× spacing (a gap equivalent to approximately 0.29 * 8 = 2.4 degrees, squares), the performance was still at 80%. But with the next-closer spacing (5× approx. 1.5 degrees, “x”), performance dropped to below 60%. Thus, the critical spacing would be considered to be near 2.4 degrees, which is approximately half the eccentricity. This procedure can be repeated for the 3 degree eccentricity, at which performance reliably drops between 5× and 2× (1.5 and 0.6 degrees, respectively) and the 7 degree eccentricity, at which performance reduces between 15× and 11× (4.35 and 3.2 degrees, respectively).

Figure 1b shows a more contemporary way to plot the data. Spacing (here, center-to-center spacing in absolute visual angle) is plotted on the x-axis, with different curves for each eccentricity (colors, as indicated by the legend). With this presentation, the curves of proportion correct vs. flanker-spacing are more readily interpretable. The appropriateness of absolute flanker spacing as a center-to-center measurement is based on the finding that critical spacing can best be understood as a zone, or the “integration field” (Pelli & Tillman, 2008), that surrounds the stimuli. The transformation is straightforward, with the center-to-center spacing being merely the edge-to-edge spacing plus one letter size.

**Figure 2.**
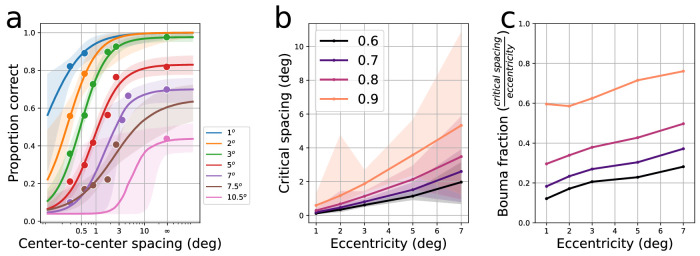
Schematic illustration of the procedure to fit crowding functions and determine the critical spacings from the data of Bouma (1970). Panel (a) shows fitted psychometric functions, with data points taken from Figure 1b. Panel (b) shows estimated critical spacings for several thresholds, defined as the abscissa value on curves in panel (a) where performance crosses threshold*asymptotic value, for thresholds shown in legend. Panel (c) plots the Bouma fraction (critical spacing divided by eccentricity) across eccentricities for each threshold.

Nowadays, a researcher would take the data as presented in Figure 1b, usually fit them with a psychometric function, and quantify the critical spacing as some arbitrary drop in performance from the asymptotic level achieved at each eccentricity. Figure 2 illustrates this procedure schematically. Panel (a) shows logistic functions fit using Markov chain Monte Carlo (Salvatier et al., 2016), assuming binomial errors (100 trials per point, as specified by Bouma). Although the approach is inherently Bayesian, we use flat priors, meaning that we do not impose biases of likely parameter values. Several notable features are seen, which we will return to throughout this article. First, the regular crowding effect is clearly visible: At each eccentricity, performance reduces as flankers are closer to the target. Note the psychometric functions are remarkably parallel (having the same slope), with the logarithmic x-axis used. On the other hand, the functions do not all asymptote at 100% when unflanked. This is the result of using the same-sized letter at each eccentricity, since the ability to identify letters (even isolated) reduces with eccentricity (Wertheim, 1891). Thus, each psychometric function is affected by both the asymptotic level (which will shift each psychometric function up or down), as well as flankers (which cause the sigmoidal reduction).

To determine the so-called “critical spacing” quantitatively requires defining an arbitrary reduction from the asymptotic (unflanked) level at each eccentricity. For example, one might define the critical spacing as the flanker spacing at which performance reduces to 50% of the asymptotic level. For the most central eccentricities (1–3 degrees), which achieve 100% performance unflanked, this corresponds to a performance level of 52% (after correcting for the guess rate of 1/25). One might also define the threshold more conservatively, to find the spacing at which flankers have a minimal effect on performance (as Bouma did); for example, one could define the critical spacing as the spacing where performance reaches 90% of the unflanked asymptotic value (or the amplitude of the psychometric function after correcting for guessing, discussed later). Figure 2b shows the estimated critical spacings derived by interpolating the threshold value from the psychometric functions in Figure 2a, for threshold relative performance levels of 60%, 70%, 80%, and 90% (as shown by legend). We have omitted the eccentricities of 7.5 and 10 degrees, as their functions were not well constrained by the data and gave unreasonably large critical spacings. As expected, critical spacing increases as a function of eccentricity, and the chosen threshold level has an impact on the critical spacing, with more conservative critical spacing thresholds (larger proportions of asymptotic values) yielding larger critical spacings. Finally, Figure 2c plots the ratio of critical spacing to eccentricity (or “Bouma fraction”). The ratios increase slightly as eccentricity increases, having a range from 0.15 to 0.3 for a threshold of 60%, 0.2 to 0.4 for a threshold of 80%, 0.3 to 0.5 for a threshold of 80%, and 0.6 to 0.7 for a threshold of 90%.

Although the commonly cited “rule of thumb” from Bouma's 1970 article invokes the pithy “half the eccentricity,” note that in a later book chapter (Bouma, 1978), Bouma suggested a proportion of “0.4 to 0.5,” which is in alignment with more recent determinations of the critical spacing (Chung et al., 2001; Levi, 2008; Pelli et al., 2004). If the rule of thumb is strictly held, Figure 2c should consist of horizontal lines, rather than rising lines. The inconsistency is likely due to Bouma's use of a constant size across all eccentricities, causing the decreases in the psychometric function amplitudes. For example, Coates et al. (2018) found that for crowding psychometric functions with different asymptotes, it was necessary to normalize curves using a *z* score transformation, instead of using a fixed threshold for critical spacing. It is possible a similar strategy would be necessary here to account for the different performance levels. In addition, Strasburger (2020) has demonstrated that Bouma's rule does not hold for small eccentricities near the fovea, which may also contribute to the non-zero slope of the lines in Figure 2c.

**Figure 3.**
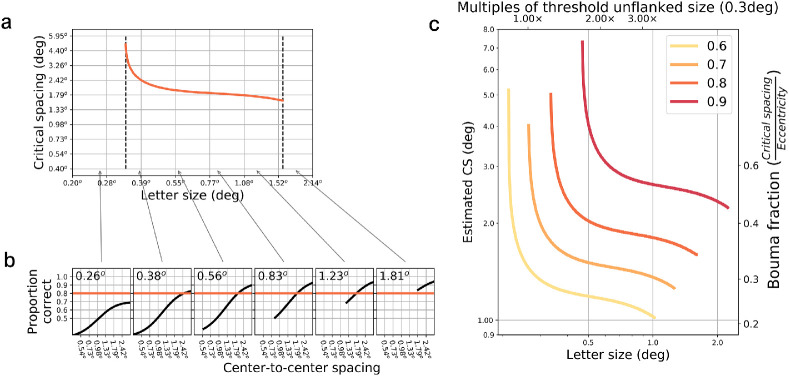
Interpolated model critical spacings for 5 degrees in the lower visual field. Each curve in (b) is a psychometric function of proportion correct versus flanker spacing at the given letter size. When letters are too small (leftmost panel), functions never reach the performance threshold defining the critical spacing (orange line: 80%). When letters are too large (rightmost panel), performance is at ceiling for all flanker spacings, eliminating crowding. Panel (a) shows the summary of critical spacings; region in middle indicates measurable sizes for crowding. Panel (c) shows estimated critical spacings (as in panel (a)) for several different critical spacing thresholds. Axis on top shows size in terms of multiples of threshold unflanked target size (75% unflanked performance), here 0.3 degrees (tested at 5 degree eccentricity in the lower visual field). Right axis shows the ratio of critical spacing divided by eccentricity (i.e., Bouma fraction).

### How to measure a crowding function

Like those shown in our reanalysis of Bouma (1970) (Figure 1b and Figure 2a), graphs showing proportion correct identification versus flanker spacing are one of the most popular ways to characterize crowding; we call the resultant curves “crowding functions.” There are some subtleties involved in effectively capturing these curves, which we summarize in the next sections.

#### Size

The use of the same-size stimuli for all eccentricities is now typically avoided, since, as seen earlier, this procedure yields curves of highly different asymptotic performance levels, possibly obscuring desired effects. Instead, stimuli are scaled to be larger in the periphery to compensate for the reduction in acuity. Typically, a threshold stimulus size is first experimentally determined that yields a reasonable baseline unflanked performance criterion, and then stimulus size is set at some multiple of the unflanked threshold size to ensure that it is not a limiting factor for the flanked conditions. For example, Toet and Levi (1992) used T targets of approximately 1.5 times each subject's unflanked threshold size (for 75% identification) at each eccentricity, resulting in targets that were identified at approximately 90% correct in isolation or with distant flankers. Then, they determined the center-to-center spatial separation between the target and the flanker that reduced identification performance down to approximately 75%.

Using a model described in the next section, it is possible to generate expected performance curves at any arbitrary letter size and spacing. The data to generate that model originated from the empirical results of Coates et al. (2013), in which observers identified Tumbling Es flanked by four Tumbling Es. In Figure 3b, we show a simulated set of curves derived from one of those observers viewing high-contrast flanked targets at 5 degree eccentricity in their lower visual field. We illustrate how critical spacing depends on letter size using an arbitrary threshold of 80% correct, which will be taken to be the performance level (as a function of flanker spacing) at which the critical spacing is defined. When the stimulus size is too small (leftmost panel), performance is generally poor, with the curve flattening at a low performance level and never reaching the threshold. Thus, it might be problematic to identify a critical spacing using a fixed performance criterion (e.g., 50% correct). When the stimuli are too large (rightmost panels), performance is too good; at all center-to-center spacings in which flankers do not physically overlap with the target, performance is at ceiling, so crowding is not observed.

From each interpolated stimulus size, critical spacings can be estimated, with the results shown in Figure 3a. Unmeasureable conditions (due to floor or ceiling performance just described) are plotted as the horizontal line near the bottom of the plot. The maximal point on the left side of the curve occurs when the corresponding proportion correct versus flanker spacing function reaches asymptote near the critical spacing threshold (e.g., panel (b), second plot from left). In these cases, the critical spacing will seem exceptionally large, so it is best to avoid asymptotic performance near the critical spacing threshold. In the middle portion of the raised part of this curve (valid sizes), critical spacings are fairly constant, with a slight decrease as letter size increases. Finally, panel (c) shows the effect of threshold on critical spacing, from a threshold of 60% (yellow curve) to 90% (red curve). Higher thresholds result in larger critical spacings, a finding that follows readily from the psychometric functions in panel (a) that are used to derive these critical spacings. In panel (c), in addition to the axes in units of visual space, we have also plotted an axis above that indicates how many multiples of the unflanked acuity size this target corresponds to, for that condition (5 degree eccentricity in the lower visual field with high-contrast stimuli). In these conditions, unflanked stimuli were identified at 75% correct when they had a size of approximately 0.3 degrees. For most thresholds, the multiples chosen in the literature (1.5x to 3x) are clearly optimal in terms of being on the flat portion of the curves. The right axis shows the ratio of the critical spacing to the eccentricity. For these parameters, the 0.5x eccentricity mentioned by Bouma is only observed with a conservative (i.e., at least 90%) criterion. More lax criteria lead to lower ratios of 0.3 to 0.4, which (as mentioned previously) are often observed in the literature.

While previous studies have shown that the critical spacing is robust to changes in target size (Tripathy & Cavanagh, 2002; Levi et al., 2002), in those cases, asymptotic performance is kept constant using some other means (such as by reducing the contrast of larger stimuli), so the findings are not directly relatable.

#### Threshold criteria

The previous section already demonstrated how the target size and threshold criteria affect the critical spacing. Specifically, Figure 3c showed that the most lax definition of threshold (60%) yields the smallest critical spacings while the most conservative (90%) yields the largest.

There are several alternative psychophysical procedures that do not rely on a fixed size target and fixed criteria, and thus are more robust to the issues described in the previous section. An approach based on proportion correct versus flanker spacing is to define a *nominal* criterion rather than a fixed performance cutoff, as we described for Bouma's data. Typically, the criterion will be defined relative to the asymptotic (unflanked) level. For example, Tripathy and Cavanagh (2002) used a reduction by 1/e of the amplitude of the psychometric function. That is, they computed the difference between the asymptotic level and the guess rate, and reduced the asymptote by a proportion of that amount. There will be little difference in the two methods if the guess rate is small (such as for Bouma's data, which used 25 letters), but for stimuli with few alternatives, such as a single rotated letter, the guess rate must be incorporated in the calculations. Alternatively, Danilova and Bondarko (2007) simply identified critical spacing as the data point with a statistically different ordinate value from the unflanked point. More generally, Coates et al. (2018) showed that when curves for foveal crowding were expressed as *z* scores of reduction from asymptotic values, curves for several different sizes collapsed onto the same template. These are just several examples to illustrate the diversity of nominal thresholds that have been used; there are countless others.

#### Abscissa: Center-to-center spacing on a logarithmic axis

Crowding functions inevitably plot proportion correct versus flanker spacing, but there are two considerations for the units of the abscissa: how to define the spacing in relation to the stimulus and whether to use linear or logarithmic units. For the first consideration, either “edge-to-edge” or “center-to-center” spacing can be used. Edge-to-edge spacing, which quantifies the spacing as the empty space between the outermost contours of targets, was used in classic studies (Bouma, 1970; Flom, Heath, et al., 1963, Flom, Weymouth, et al., 1963) and others, but is less used nowadays except for more clinical studies or those evaluating contour interaction. As we showed, the critical spacing decreases slightly as letter size is increased, but the decrease would be much more drastic using the edge-to-edge definition of critical spacing. Conceptually, the center-to-center concept matches an elliptical zone extending from the centers of targets.

On the other hand, there is no consensus in the literature about whether linear or logarithmic units of visual angle are preferable for plotting flanker spacing. Here we make the claim that a logarithmic measure is definitely more appropriate for the flanker spacing abscissa of psychometric functions for several reasons. First, we have found that crowding functions from disparate conditions often line up (have the same slope but different 50% points) but only when presented on a logarithmic axis, which was observed earlier when analyzing Bouma's data. Furthermore, Figure 9, to be described in greater detail later, presents data collected from the same observer under several different stimulus conditions. The left and right panels show results from two different stimulus durations, with additional stimulus manipulations of varied target and flanker types as well as different visual field locations. Clearly, the same slope could be used to fit all of the curves, but this is highly dependent on the abscissa being in logarithmic coordinates; with linear coordinates, the rightmost curves would appear to be flatter. A corollary of the use of a logarithmic axis for flanked distance is that critical spacing modulations (including interactions from multiple stimulus manipulations) should be considered multiplicative. For example, the ratios between the four conditions on the left panel (short duration) are similar to the ratios of the four conditions on the right panel (long duration), despite the *differences* within the conditions for each duration being very different. This perspective may yield an alternative explanation of other nonadditive effects of combined stimulus manipulations observed in crowding (Soo et al., 2018).

### Alternate methods: Contrast thresholds and flanked acuity

Besides collecting proportion correct versus flanker spacing curves, there are a variety of other ways to measure the critical spacing of crowding. Specifically, experiments may also track some other measure besides identification performance. For example, threshold contrast to identify optotypes of a particular size and flanker spacing can be used (Strasburger et al., 1991; Chung et al., 2001; Pelli et al., 2004). The advantage is that size and spacing can be held constant while the dependent variable (e.g., contrast) varies. This method has been previously used, with the critical spacing taken to be the point at which the threshold measure (e.g., contrast) becomes detectably greater than the baseline (unflanked) level, again using some arbitrary criterion, such as a doubling of thresholds (Levi et al., 2002), fitting a multiline curve (Chung et al., 2001; Pelli et al., 2004), or simply an apparent divergence of crowded curves from uncrowded curves (Strasburger et al., 1991). The two-line fit method involves fitting two separate lines to threshold versus flanker spacing curves. The typical pattern observed is a portion that is flat and unaffected by flankers beyond a critical spacing (suitably large flanker spacings behave like unflanked trials), and a portion where thresholds reduce when the flankers get closer to the target, indicating a clear influence of crowding on performance. Identifying these two lines and finding their intersection is akin to using a highly conservative criterion (i.e., near 100%) for proportion correct versus flanker spacing. Thresholds found with these alternative methods (whatever the fitting criteria) are presumed to be related to those measured using proportion correct versus flanker spacing curves, which we illustrate below.

One particularly useful variation is to modulate the size of targets and flankers while keeping the *nominal* spacing (spacing in terms of letter widths apart) fixed, which henceforth will be referred to as the flanked acuity paradigm. This size threshold measurement, which is similar to the measurement of acuity with a clinical letter chart, has several advantages, both theoretical and practical. For example, with an adaptive procedure such as a staircase on flanked size, testing can determine a threshold with little prior knowledge about its value, permitting use in diverse populations. A single staircase measuring flanked acuity at some nominal spacing will vary the target (and flanker) size, which will also change the absolute target/flanker distance. Thresholds measured in this way follow a very regular pattern, with a predictable trade-off of threshold size versus spacing for small spacings, and an independence of threshold on spacing for large spacings. This pattern is observed in the normal periphery (Coates et al., 2013; Song et al., 2014; Chung, 2014), including with low-contrast stimuli (Coates et al., 2013), in amblyopia (Song et al., 2014), and in central vision loss (Chung, 2014). With assumptions about the curves, in theory, only two points need to be measured: one on the crowding portion of the curve and one in an unflanked condition, making this an efficient method of estimating the critical spacing.

**Figure 4.**
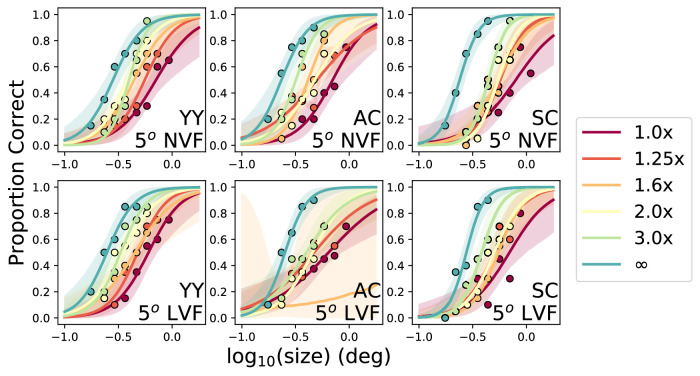
Psychometric functions derived from a control experiment from Chung (2014). Observers are shown in columns and visual field (nasal [NVF] or lower [LVF]) in rows. Ordinates show proportion correct performance identifying flanked letters at different *nominal* spacings (given by colored points and curves), with letter size indicated on the abscissa. Absolute center-to-center spacing can be determined by multiplying the abscissa by the nominal spacing. Shaded regions indicate 95% confidence intervals from 1,000 Monte Carlo fits.

A theoretical advantage of this method is that the psychometric functions collected are everywhere “well behaved,” varying from a minimal (unflanked) threshold to a maximum (flanked) threshold. The issues seen earlier with proportion correct versus absolute flanker spacing (failure to reach a sufficient performance asymptote, or a ceiling effect) are absent, and often an adaptive procedure will be used to determine the threshold size. Importantly, we have found that the slope of the psychometric function does not vary with this procedure, at least across contrast, eccentricity, and nominal flanker spacings (Coates et al., 2013). These data were collected via a staircase procedure, with raw data refit to determine the slopes, and thus might be regarded with some reservations. An alternative set of data was collected using a constant stimuli paradigm, in a control experiment reported by Chung (2014). In this experiment, subjects identified flanked lower case Times Roman letters with nominal spacing stimuli; a block at one nominal spacing comprised different stimulus sizes, which changed the absolute spacings at the same time. However, these stimuli were shown in a constant stimuli paradigm to yield full psychometric functions. Figure 4 shows the psychometric functions for three subjects at 5 degree eccentricity in both the lower and nasal visual fields. Importantly, despite differences in the overall performance at the different visual field locations (shown in different panels) and with the different nominal spacings (colors points and lines), the same slope can be used for all these psychometric functions; a model with only a horizontal shift in each psychometric function can adequately describe all these data.

### Flanked acuity model

**Figure 5.**
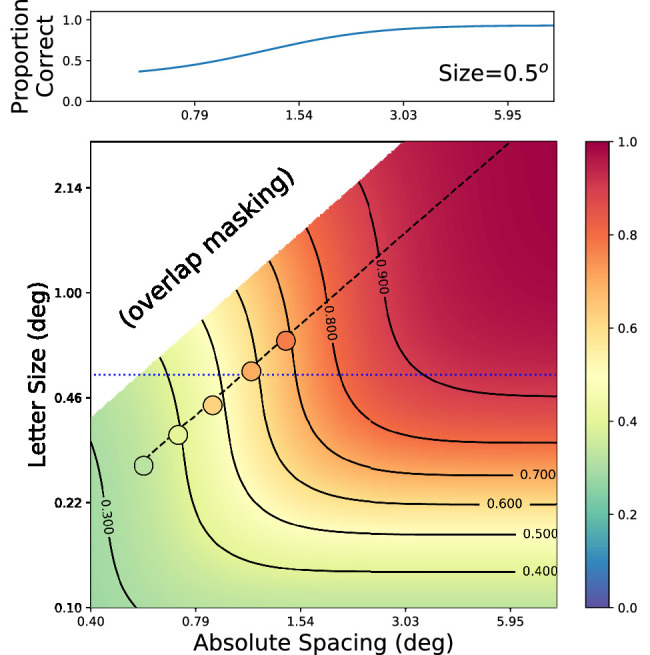
Extrapolated landscape of flanked performance from model derived from Coates et al. (2013), with observer JMC at 5 degree eccentricity in the lower vision field. Each point on the central two-dimensional grid indicates the expected proportion correct (indicated by color, see color bar) for identifying a flanked letter of a certain size (y position) crowded by flankers at some absolute spacing (x position). Curved lines indicate iso-performance curves. The angled dashed line indicates points corresponding to one nominal spacing (2×). Filled circles indicate empirical data from a different experiment, shown in Figure 4, Subject SC, lower visual field, 2× spacing. Upper curve shows the inferred typical crowding function for a particular letter size (0.5 degrees, horizontal dotted line in main plot) flanked at different absolute spacings.

It was stated earlier that results determined from flanked acuity experiments described in the preceding section can be related to results from the alternative method measuring proportion correct. Both paradigms measure the ability to identify flanked targets; they simply modulate stimulus parameters in different ways. To illustrate this, Figure 5 shows a two-dimensional grid that shows expected proportion correct at 5 degree eccentricity for combinations of stimulus size and spacing (data derived from one observer from the study by Coates et al., 2013). Hot colors represent easier targets (high proportion correct) while cool colors indicate harder targets (low proportion correct). Letter size is on the y-axis while absolute center-to-center flanker spacing is on the x-axis. The psychometric functions seen earlier (in Figure 3) are horizontal slices of this plot. When letters are too small (bottom of plot), performance never gets to a sufficient level, even for wide flanker spacings (bottom right side). When letters are too large (top), there is a large region of center-to-center spacings that would be limited by physical overlap of the flankers and targets (white region), consideration of which is outside the scope of this article. With large letters, the spacings beyond the overlap region (upper right) are likely to yield ceiling levels of performance, precluding a measurement of critical spacing. The critical spacing, given an arbitrary threshold criterion, can be simply read off from the desired iso-performance line. In fact, the plots shown in Figure 3 were derived in precisely this way.

As described earlier, studies that use stimuli of a certain size will attempt to stay in the middle of the range and typically have used the method of constant stimuli to vary flanker spacing to capture crowding functions. The example at the top of Figure 5 shows a psychometric function derived in this way, which corresponds to the horizontal dotted line in the main plot. The flanked acuity paradigm, on the other hand, varies target/flanker size and absolute spacing at a fixed nominal spacing at the same time, which translates to a diagonal line in this plot. The black dashed line shows how a stimulus with a fixed nominal spacing varies along the two dimensions, at a nominal center-to-center spacing of 2 times the letter size. A staircase procedure like that used in Coates et al. (2013) will move along this diagonal line to determine a threshold size level at that nominal spacing. Overlaid on the plot as filled circles are empirical results from one subject (Subject SC) at a nominal spacing of 2× from the study described earlier (Figure 4, from Chung [2014]). The color of each circle indicates the proportion correct in a method of constant stimuli paradigm. The proportion has been converted to account for the different guess rates of the two studies (1/26 for the data from Chung [2014], 1/4 for the data from Coates et al. [2013]). Note that despite the differences in the stimuli (Tumbling Es vs. lowercase letters) and the paradigms, there is reasonable agreement between the empirical results and predictions from the model. The model itself will be the topic of a future paper, but (briefly) is based on few parameters, comprising two functions:

(1) First, a base psychometric function is necessary to describe nominal flanked acuity, like those shown in Figure 4, with a fixed slope across all conditions (including eccentricity and nominal flanker spacing). These functions describe proportion correct along the diagonal lines of nominal flanked acuity.

(2) Then, a function must capture the effects of flankers at a given eccentricity on the 50% points of Figure 4, which gives the iso-performance curves in Figure 5 their kinked shape. This function is a hinged line: a line with two discrete connected portions having different slopes. For closely flanked nominal spacings, the function is inversely proportional to flanker spacing (increased flanker spacing yields lower values), which matches Figure 4, where the functions are shifted to the left for wider spacings. However, at some nominal spacing, flankers no longer affect thresholds (being outside the crowding zone), so this function flattens to zero slope, and the resultant psychometric functions overlap. There have been several proposals for this function, including a hinged line with a crowded slope of -1 inside the critical spacing and a slope of 0 outside (Chung, 2014; Song et al., 2014; Coates et al., 2013), which translates to purely right-angle iso-performance curves in Figure 5. Previous work has also proposed a parabola or reciprocal line on arbitrarily scaled axes (Latham & Whitaker, 1996; Gurnsey et al., 2011) and finally a hinged line in units of cortical distance (Coates et al., under review). Here, we found that on an abscissa of $\frac{size}{unflanked\_size^{2}}$, a hinged line captured the entire set of results shown in Figure 5, with a slope in the crowding portion of approximately −0.8.

## Effects of stimulus parameters on critical spacing

### Size

The impact of size on critical spacing has been mentioned earlier, in that sizes outside some critical range cannot be used to determine critical spacing since they lead to poorly behaved psychometric functions. In agreement with prior studies, using a size inside the acceptable range will generally lead to consistent results: the curves in Figure 3c are fairly flat, except when the psychometric function's upper asymptote is too near the threshold. This demonstration supports the relative independence of the extent of crowding on target size as a distinctive criterion of crowding (Pelli et al., 2004). The small decrease in critical spacing with larger target sizes we report is likely due to differences in asymptotic performance levels.

### Contrast

It has been known since at least Kooi et al. (1994) that differences in contrast between the target and flanker play a role in crowding. In that study, mixtures of contrasts yielded variable results, likely including the effects of target-flanker similarity as well as weaker signals from low-contrast target and flanker letters. There was a hint that low-contrast targets and low-contrast flankers had larger interaction zones than those of high-contrast targets, but this was not stated explicitly. A similar effect could be deduced from the flanked contrast thresholds of Strasburger et al. (1991, Figure 6). Most of the subsequent studies have investigated the impact of *different* contrasts between the target and its flankers (Chung & Mansfield, 2009; Rashal & Yeshurun, 2014), with the exception of Coates et al. (2013), who looked specifically at the more general role of contrast in crowding by testing a variety of flanker spacings and eccentricities, with yoked target and flanker contrasts, from a high contrast of 99% down to a low contrast of 2.7%.

**Figure 6.**
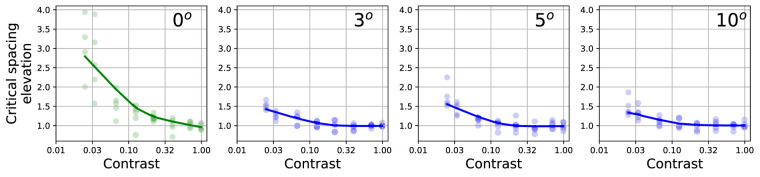
Effect of yoked target and flanker contrast on the critical spacing at the fovea and up to 10 degree eccentricity (from left to right), replotted from the data of Coates et al. (2013). The ordinate indicates the ratio of the critical spacing measured at each contrast relative to the critical spacing measured at high contrast. Lines represent LOWESS fits.

Coates et al. (2013) determined size thresholds using a staircase procedure and the above mentioned flanked acuity method across conditions of various contrasts, eccentricities, and nominal flanker spacings. A main finding from this study was that lower contrast stimuli led to slightly larger crowding zones, as summarized in Figure 6. The increase in critical spacing is especially marked in the fovea, increasing to 3 times the high-contrast value for contrasts below 3%. In the periphery, the effect is less marked, with a corresponding enlargement of only approximately 1.4 times the high-contrast critical spacing. The greater impact of contrast foveally is likely due to the fact that foveal thresholds are more limited by the resolution needed for individual character recognition, whereas peripheral thresholds are more limited by crowding, which is less affected by contrast. The effect of contrast would be in general agreement with findings from neuroscience that have reported enlarged receptive fields for cortical neurons for low-contrast stimuli (Kapadia et al., 1999; Sceniak et al., 1999; Cavanaugh et al., 2002).

**Figure 7.**
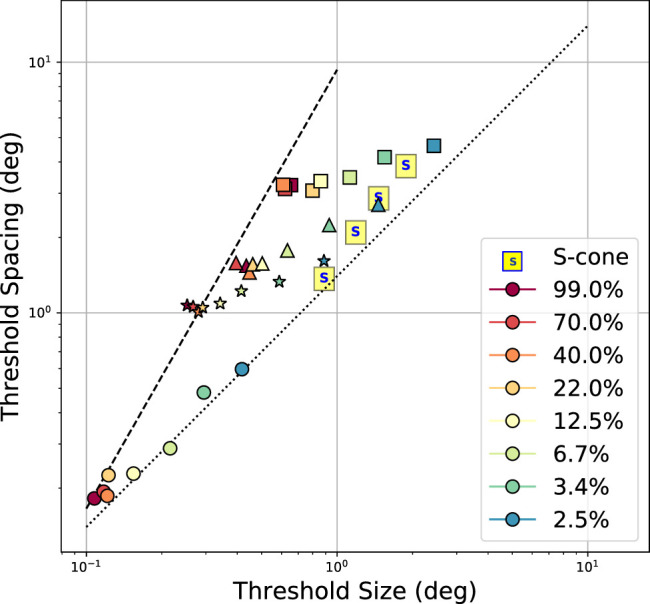
Threshold critical spacing plotted versus threshold unflanked letter size for results from flanked acuity experiments with different contrasts (Coates et al., 2013; Coates & Chung, 2016). Colors indicate stimulus conditions (reduced target/flanker contrast or S-cone isolation condition). Symbols indicate target eccentricity (0 [circles], 3 [stars], 5 [triangles], 10 [squares] degrees) for contrast experiments. Dotted and dashed lines indicate theoretical predictions from Song et al. (2014) for foveal and peripheral stimuli, respectively. Dotted line is near unity, where threshold size and threshold spacing change in tandem, indicating that blur is the limiting factor. Dashed line is steeper, indicating that in peripheral vision, critical spacing changes more steeply with eccentricity than threshold size. Lowering stimulus contrast causes a shift between the two regimes.

Another way to plot the results is to present threshold critical spacing versus threshold unflanked letter size at each condition, such as shown in Figure 7. The different symbols indicate eccentricity (circles: fovea, stars: 3 degrees, triangles: 5 degrees, squares: 10 degrees) for the contrast experiments (Coates et al., 2013). The four S-cone points (Coates and Chung, 2016) indicate results from 0, 3, 5, and 8 degrees eccentricity, respectively, from lowest to highest on the plot. The dotted and dashed lines show theoretical predictions from Song et al. (2014) for foveal and peripheral stimuli, respectively. In the fovea, both the threshold size and critical spacing of stimuli change proportionally (dotted line), meaning that size limitations (driven by blur) dominate—the critical spacing increases only to avoid overlap between target and flanker. On the other hand, in the periphery, the critical spacing changes much faster with eccentricity than the threshold (unflanked) letter size, which is indicated by the steep dashed line (accelerated dependency). As seen in the graph, higher contrast stimuli (22% or greater stimuli; hotter colors) fall near the dashed line, denoting previous peripheral results (which used only high-contrast stimuli). As contrast is reduced (cooler colors), the points shift both to the right as well as upward, indicating an increase in both threshold size and flanker spacing. The move away from the dashed line toward the dotted line indicates a transition from a crowding-limited regime to a more blur-limited regime. At the lowest contrast, a line connecting the points would be near 45 degrees and close to the blur-limited line, reinforcing the finding that lowering the contrast affects threshold resolution more than it affects crowding.

### Duration

Several studies have reported that critical spacing becomes smaller with longer stimulus duration. This result is consistent with the coarse-to-fine notion of spatial analysis with exposure duration (Watt, 1987). When a stimulus first appears, the visual system is more sensitive to the coarser, or the lower spatial-frequency, information. With time, the sensitivity shifts to the finer, or the higher spatial-frequency, information. This shift in spatial scale with time translates into an initially larger integration field, or in relation to crowding, a larger critical spacing, which becomes smaller with longer stimulus duration. To explore whether there is a general relationship governing the change in critical spacing with stimulus duration, we reviewed over a dozen studies that measured critical spacing using optotypes as stimuli in normal peripheral vision. All these studies used proportion correct of target recognition as the performance measurement, and thus there is no concern about comparing studies using different performance measurements. In about half of these studies, the target was flanked by four flankers in the cardinal directions relative to the target. For the rest of the studies, only two flankers were presented with the target, along either the radial or the tangential direction. Because different eccentricities were tested in different studies, we will compare the Bouma fraction as a function of eccentricity instead of the physical size of critical spacing.

**Figure 8.**
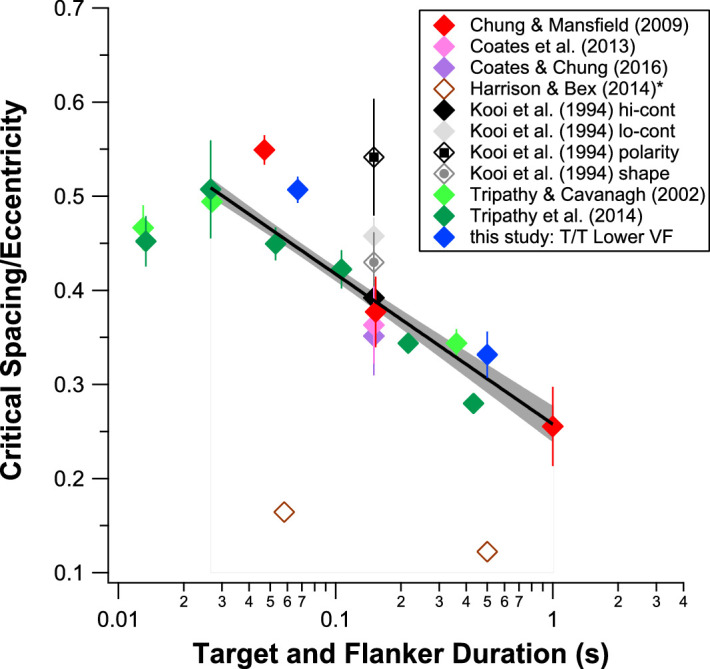
Ratio of critical spacing to eccentricity (Bouma fraction) is plotted as a function of target and flanker presentation duration for several studies (see legend for details), in which a target was surrounded by four flankers. Many of these studies tested a number of stimulus conditions, and our criteria for data to be included are listed in Table 1. The black line represents the best-fit regression line to the set of data on semi-log axes, excluding those of Harrison and Bex (2014). The slope of this line is −0.16 ± 0.02, with the ±95% confidence bands represented by the shaded region. A similar result was obtained when we separately fit a line to the aggregate data that included data from studies using only two radial flankers, in addition to the set of data shown here (slope of the resultant best-fit line = −0.17 ± 0.01; see text for details).

It has been shown that performance of recognizing an object declines with the number of flankers (Põder & Wagemans, 2007; Levi & Carney, 2009), at least for Gabor or line stimuli (except in the extreme conditions when the flankers can be grouped together to release the target from crowding; Levi & Carney, 2009; Livne & Sagi, 2010; Manassi et al., 2012; Saarela et al., 2009), arguing against the simplest models of crowding (Herzog et al. 2015). For letter or optotype stimuli, Pelli et al. (2004) showed that the crowding effect was similar with two or four flankers. Here, we will first summarize how the Bouma fraction changes with target and flanker duration for data obtained with four flankers. Figure 8 plots Bouma fractions as a function of target and flanker duration. All the data plotted in this figure were obtained when the target and flankers coexisted in time, and thus the target and flanker durations were identical. Two studies (Coates et al., 2013; Coates & Chung, 2016) tested several eccentricities. For each of these studies, a one-way ANOVA showed that there was no significant difference in the Bouma fractions at different eccentricities; therefore, these values were averaged to yield one single value for each study (only one duration was used in these studies). Clearly, with the exception of the shortest duration, the Bouma fraction appears to fall with increased duration. To quantify the relationship, we fit a line to the data points on the semi-log plot. Because the two data points for Harrison and Bex (2014) (unfilled brown symbols) appeared to be outliers, they were excluded for the fitting (we will return to this later). For the rest of the data points (all filled symbols), we iteratively excluded data points starting from the shortest duration (13 ms) to search for the best-fit line with the lowest reduced chi-square. The best-fit line, as shown in black, excludes only the 13-ms data points. The slope of the line is −0.16 ± 0.02, meaning that for every log-unit increase in duration, the Bouma fraction is reduced by 0.16. Wallace et al. (2013) previously reported a slope of −0.27 when both the Bouma fraction and duration were plotted on log-log axes. Following their method of data fitting, the slope of our data, on log-log axes, is −0.20 ± 0.03, still a bit shallower than the value of Wallace et al. (2013). However, we included more studies in our analysis and Wallace et al.'s fitted slope was heavily weighted by their own data, which also included data obtained using an artificial scotoma (but excluded from our analysis here; see Table 1 for details).

**Table 1.** Experimental details of the studies surveyed, with data plotted in Figure 8 and in Appendix Figure A.1. *Notes*: LVF = lower visual field; RVF = right visual field; NVF = nasal visual field; T-F = target-flanker; VF = visual field; LNVF: lower-nasal visual field; R/T: radial/tangential.

Study	Eccentricity/VF	Target	Flankers (including number)	Target & flanker size	T-F orientation with respect to fixation	Duration (s)	Task	Performance measurement	Method of defining threshold	Comments
Chung et al. (2001)	5${}^{\circ }$ LVF	Lowercase letters (Times Roman)	Same as target, 2	1.1${}^{\circ }$ (2× the single-letter size threshold)	Tangential	0.15	Identify the target letter	Contrast threshold, defined as contrast that yields 50% correct identification	Critical spacing is defined based on the intersection point of the two-line fit to the data of contrast thresholds as a function of T-F separation	Only the unfiltered letter condition
Chung et al. (2007)	10${}^{\circ }$ LVF/NVF	Lowercase letters (Times Roman)	Same as target, 2	2.7${}^{\circ }$ (6× larger than the first-order single-letter acuity)	Radial/tangential	0.15	Identify the target letter	Contrast threshold, defined as contrast that yields 50% correct identification	Critical spacing is defined as the T-F separation that corresponds to 2 *SD* from the peak of the Gaussian fit to the data of contrast threshold elevation (ratio of flanked to unflanked contrast threshold) vs. T-F separation	Only the 111 condition
Chung and Mansfield (2009)	10${}^{\circ }$ LVF	T	T, 4	0.5${}^{\circ }$ whole letter size, fixed across conditions	Radial/tangential	0.047, 0.153, 1	Identify orientation of target T	Identification accuracy, vary T-F separation	62.5% from the psychometric function (proportion correct vs. spacing)	Only the BB and WW conditions
Chung (2014)	5, 10${}^{\circ }$ LVF, NVF	Lowercase letters (Times Roman)	Same as target, two flankers along the horizontal dimension	Size varied with nominal spacing	Radial for NVF, tangential for LVF	0.15	Identify the target letter	Size threshold for a given nominal letter spacing	Threshold defined as 50% (after correction for guessing), but critical spacing defined based on fitting size threshold vs. spacing data	Only normal adult control data included
Coates et al. (2013)	0, 3, 5, and 10${}^{\circ }$ LVF	Tumbling E	Same as target, 4	Size varied with nominal spacing	T-F separations for the four flankers were yoked, thus could not separate out the R/T direction	0.15	Identify orientation of target E	Size threshold for a given nominal letter spacing	Threshold defined as 79% correct identification, but critical spacing defined based on fitting size threshold vs. spacing data	
Coates and Chung (2016)	0, 3, 5, and 8${}^{\circ }$ LVF	Tumbling E	Same as target, 2 or 4	Size varied with nominal spacing	Two flankers: tangential four flankers: T-F separations yoked, thus could not separate out the R/T direction	0.15	Identify orientation of target E	Size threshold for a given nominal letter spacing	Threshold defined as 79% correct identification, but critical spacing defined based on fitting size threshold vs. spacing data	Only the LC4 condition
Harrison and Bex (2014)	9${}^{\circ }$ RVF	U (4-orientation)	A set of 17 letters (excluding U)	0.5${}^{\circ }$ whole letter size	T-F separations for the four flankers were yoked, thus could not separate out the R/T direction	0.058, 0.5	Identify orientation of target U	Identification accuracy, vary T-F separation	62.5% from the psychometric function (proportion correct vs. spacing)	Only the T/F synchronous condition
Kooi et al. (1994)	10${}^{\circ }$ LVF	T (bar-width 1/5 of whole letter size, 0.5${}^{\circ }$), four orientations	In general, same as target but different parameters were examined (e.g., color, polarity, disparity, luminance)	0.5${}^{\circ }$ whole letter size, fixed across conditions	T-F separations for the four flankers were yoked, thus could not separate out the R/T direction	0.15	Identifying the orientation of the target T	Identification accuracy	62.5% on the psychometric function	Only the following conditions are included: same polarity, same shape, and same contrast (83% and 29%)
Pelli et al. (2004)	One expt examined different ecc up to 25${}^{\circ }$, but the rest of the conditions only tested at 4${}^{\circ }$ (right VF)	Chosen from 10 Sloan letters	Same as target, most of the times two flankers, except for one expt when they examined the number of flankers	In the expt of examining ecc, target/flanker size was 1${}^{\circ }$ (fixed across ecc). In the expt examining fovea vs. 4${}^{\circ }$ ecc, target/flanker size was 0.32${}^{\circ }$. In the expt examining size, target size ranged between 0.32 and 2${}^{\circ }$.	Two flankers: radial	0.2	Identify the target letter	Contrast threshold for identifying the target letter	Critical spacing estimated from a clipped line function fitted to the data of contrast threshold as a function of T-F separation	Only from their Figures 3, 4, and 5c
Pelli et al. (2007)	5 to 12${}^{\circ }$, LVF, right VF, lower-right VF	10 Sloan letters	Same as target, 2	Various letter sizes used (three per testing location); see their Table 1	Radial/tangential/45${}^{\circ }$/135${}^{\circ }$	0.2	Identify the target letter	Identification accuracy, vary T-F separation	Letter separation such that identification accuracy was 80%	Data were only from one subject (KAT)
Toet and Levi (1992)	0, 2.5, 5, and 10${}^{\circ }$ LVF, NVF, LNVF	T (thin lines making up Ts), only upward or downward	Same as target	1.5× the resolution threshold (acuity) for a single T, determined separately for each ecc	Radial/tangential	0.15	Identifying the orientation (up/down) of the target T	Measure the size threshold first, then use this size threshold to determine the critical spacing	75% on the psychometric function (50% correct after correction for guessing)	No interaction when only one flanker was used (contrary to Bouma's)
Tripathy and Cavanagh (2002)	9.2${}^{\circ }$ LVF	T	Square thetas, same size as target T	0.37${}^{\circ }$ to 1.85${}^{\circ }$, varied letter size when duration was changed	T-F separations for the four flankers were yoked, thus could not separate out the R/T direction	Varied, depending on stimulus size (0.013, 0.027, 0.36 s)	Identifying the orientation of the target T	Identification accuracy	Critical spacing defined as the point on the fitted cumulative normal curve that corresponds to a drop in the percent correct response by a factor 1/e from the unflanked performance	Only luminance condition included
Tripathy et al. (2014)	10${}^{\circ }$ LVF	T	Square thetas, same size as target T	Size and luminance varied to equate for visibility, but sizes only ranged between 0.5${}^{\circ }$ and 1${}^{\circ }$	T-F separations for the four flankers were yoked, thus could not separate out the R/T direction	Varied, from 0.013 to 0.427 s	Identifying the orientation of the target T	Identification accuracy	Critical spacing defined as the point on the fitted cumulative normal curve that corresponds to a drop in the percentage of correct responses by a factor 1/e from the unflanked performance	Data from both expts included. Experiment 1: only included Figure 3 left (either keeping target size constant but varying luminance to equate for visibility or keeping luminance the same while varying size). Experiment 2 only the same-polarity data were used (Figure 4, left); both target size and luminance were varied to equate for visibility when duration changed
Wallace et al. (2013)	6${}^{\circ }$ LVF	Lowercase letters (Arial)	Same as target, 2, contrast of flankers fixed at 30%	1.5× the unflanked acuity size	Radial	0.25 vs. unlimited	Identify the target letter	Contrast threshold as a function of center-to-center T-F separation	Critical spacing threshold defined based on the clipped line function	

### Target-flanker similarity, choice of specific letters, and visual field location

In Figure 8, the critical spacings reported by Harrison and Bex (2014) are very small, resulting in very small Bouma fractions (0.12-0.16, depending on the target duration). In their study, they presented a target letter U at 9 degree eccentricity in the right visual field at one of four possible orientations, which was flanked by four upright letters, chosen randomly from a set of 17 uppercase letters. The task of the subjects was to indicate the orientation of the target letter U. Although there were several different temporal conditions included in their study, we only used their data obtained when the target and flankers were presented concurrently, providing data for 58- and 500-ms presentations. They varied the target-flanker separation and measured the proportion correct of identifying the orientation of the letter U. Critical spacing was defined as the target-flanker separation corresponding to 50% of correct identification (after correction for guessing), similar to the criterion adopted by Chung and Mansfield (2009) and Kooi et al. (1994). At first glance, all these details of the experimental design and the choice of stimulus seemed to be similar to those of the other studies that we analyzed in Figure 8. What then could have accounted for their much smaller critical spacings observed?

In an attempt to reconcile the finding of Harrison and Bex (2014) with the other studies cited in Figure 8, we identified several experimental parameters in their study that were different from the others cited in Figure 8. First, although their target was also a single letter that could be presented in one of four orientations, unlike the other studies, their flankers were not the same as (Chung & Mansfield, 2009; Coates et al., 2013; Coates & Chung, 2016; Kooi et al., 1994) or highly similar to (Tripathy & Cavanagh, 2002; Tripathy et al., 2014) their target. Instead, they used a set of 17 letters presented in their upright positions as flankers. Some of these flankers do not seem to share any features with the target letter U (e.g., A) while other flankers seem to share some similar features with the target letter (e.g., M, E). The complexity of these flankers also varied (e.g., the letter I has a lower complexity than the target U, whereas M has a higher complexity than the letter U). Similarity and complexity differences between a target and its flankers are known to affect crowding (Bernard & Chung, 2011; Kooi et al., 1994). An increase in similarity between a target letter and its flankers leads to increased letter identification errors (Bernard & Chung, 2011; Chastain, 1982; Nazir, 1992) and/or increased critical spacing (Kooi et al., 1994), which may be exacerbated by confusions between the target and flanker (Strasburger, 2020, p. 28).

Besides the target-flanker similarity, the choice of the letter U as the target was also unconventional, since most studies used Tumbling E, Landolt C, or the letter T for a four-orientation task. However, we do not know of any studies that have investigated how the choice of a specific letter as the target affects critical spacing. On one hand, critical spacing has been shown to be invariant with stimulus types (Martelli et al., 2005; Wallace & Tjan, 2011). On the other hand, different letters are known to have different legibility (Ferris et al., 1993; Reich & Bedell, 2000; Sloan, 1951); therefore, conceivably, the critical spacing could differ depending on the specific selection of a target letter. Finally, all the studies cited in Figure 8, with the exception of Harrison and Bex (2014), were performed in the lower visual field. Visual capabilities such as resolution and contrast sensitivity are known to vary with visual field location (Wertheim, 1891; Phelps, 1984; Rijsdijk et al., 1980; Himmelberg et al., 2020). Visual field-dependent changes in the critical spacing for crowding have also been noted (Toet & Levi, 1992; Greenwood et al., 2017), so it is plausible that this effect may have contributed to the small critical spacing seen in Harrison and Bex (2014).

To investigate whether or not the much smaller critical spacings reported by Harrison and Bex (2014), when compared with other studies cited in Figure 8, were the result of target-flanker dissimilarity, the specific choice of their target letter U, and/or the use of the right visual field, we determined the critical spacing for several conditions that differed in their combinations of target and flankers. Our goal was to first replicate the result of Harrison and Bex (2014) and then systematically evaluate the contribution of each of the aforementioned factors. Consequently, we followed closely the experimental details of the study by Harrison and Bex (2014). Specifically, we tested four subjects at 9 degree eccentricity in their right visual field (unless otherwise stated), using the same font (Sloan letters) and letter sizes (0.5 × 0.5 degrees) as in Harrison and Bex (2014). Two stimulus durations were tested: 67 ms and 500 ms, closely matching those used by Harrison and Bex (2014) (58 ms and 500 ms). The first condition (U/L) we tested was almost the exact replica of Harrison and Bex (2014), with the letter U being the target and flanked by four randomly chosen letters from the same set of flankers as in Harrison and Bex (2014). The second condition (U/U) differed from the first one in that the flankers were also letter Us, with each one presented randomly in one of four orientations. Differences in critical spacing between the U/L and U/U conditions would be attributable to target-flanker similarity. In the third condition, letter Ts replaced letter Us as both the target and flankers (T/T condition). Each of these Ts could be presented in one of four orientations. A comparison in results between the U/U and T/T conditions would indicate whether critical spacing depends on the specific choice of letters as target and flankers. Last, we repeated the T/T condition, but this time, in the lower visual field instead of the right visual field to evaluate whether critical spacing depends on the visual field location.

For each combination of condition and duration, we used the method of constant stimuli to present the target and its flankers at eight target-flanker separations, along with the unflanked condition. Each separation was repeated 20 times in each block so that we could measure the proportion correct of identifying the orientation of the target letter as a function of target-flanker separation. Each subject completed between two and four blocks of trials for each condition (combination of target and flankers × presentation duration). The experimental protocol was approved by the Institutional Review Board at both the University of Houston and the University of California, Berkeley, and was conducted in accordance with the Code of Ethics of the World Medical Association (Declaration of Helsinki). All four subjects gave written consent before data collection commenced.

**Figure 9.**
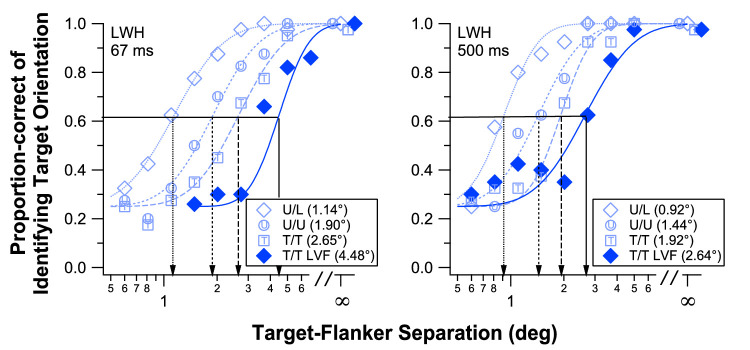
Proportion correct of identifying the orientation of the target letter presented at 9 degree eccentricity is plotted as a function of target-flanker separation for subject LWH, for the four target/flanker combinations, and for the two target and flanker durations. The infinity symbol on the x-axis represents the unflanked condition. Symbols in the plot represent the average performance across different blocks of the same condition. Smooth curves represent the best-fit cumulative Gaussian functions. Critical spacing is defined as the target-flanker separation corresponding to a proportion correct of 0.625 on the cumulative Gaussian function and is given for each condition in parentheses in the legend in each panel.

For each subject, data were combined across different blocks of the same condition and were subsequently fitted with a cumulative Gaussian function. Critical spacing was defined as the target-flanker separation that corresponded to 62.5% correct (50% after accounting for the chance level) on the cumulative Gaussian function. Figure 9 summarizes the performance of one of our naive subjects for the four testing conditions. As mentioned earlier, despite the differences in the target-flanker types and the different visual field locations, the four psychometric functions in each panel exhibit very similar slopes. Note that the psychometric functions given in this figure did not have their slopes constrained or fixed at a specific value.

**Figure 10.**
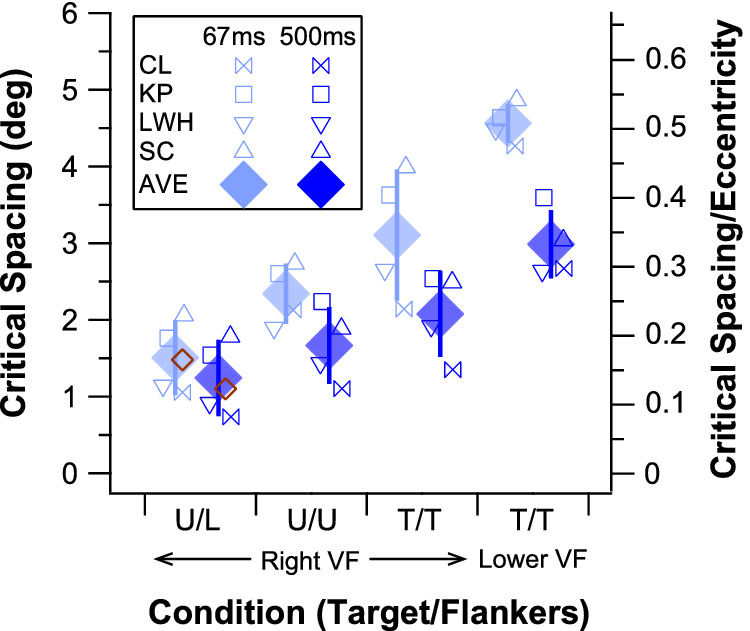
Summary of the effect of stimulus parameters on critical spacing in new experiments. Critical spacing is plotted on the left y-axis and the ratio of critical spacing to eccentricity (Bouma fraction) is plotted on the right y-axis. Filled symbols represent the group-averaged values, with error bars representing ±1 *SE*. Unfilled blue symbols represent individual subjects’ data and unfilled brown diamonds represent the data of Harrison and Bex (2014).

Figure 10 compares the critical spacing for the four combinations of target and flankers. Individual small unfilled symbols represent the critical spacings for individual observers while the filled diamonds represent the averaged critical spacing for the four observers. The critical spacings reported by Harrison and Bex (2014) are plotted (brown unfilled diamonds) alongside our data for the U/L condition, showing that our data are in excellent agreement with theirs. This figure clearly demonstrates that the critical spacing depends strongly on the letters used for the target and its flankers, as well as the target duration. A linear mixed-effects model with the combination of target and flanker letters and stimulus duration as fixed effects and observers as a random effect confirmed the significant main effects of target and flanker letters *F*(3, 21) = 96.8, *p* < 0.0001 and duration *F*(1, 21) = 106.3, *p* < 0.0001 on critical spacing, as well as a significant interaction effect between the two factors *F*(3, 21) = 10.6, *p* = 0.0002. In general, critical spacing is smallest when a target U was surrounded by other letters (U/L), the configuration used by Harrison and Bex (2014). When the flankers were replaced by letter Us (U/U), the critical spacing became 1.55× and 1.34× larger, for the 67-ms and the 500-ms condition, respectively. We attribute the larger critical spacing obtained for U-flankers compared with random-letter flankers to target-flanker similarity. Previous studies (Kooi et al., 1994; Bernard & Chung, 2011; Chastain, 1982; Nazir, 1992) reported that increased target-flanker similarity leads to increased identification errors. Here, for a range of target-flanker separations (except for the very small ones when performance was at chance or the very large ones when performance was at ceiling), identification errors were higher for the U/U than the U/L condition (see Figure 9), leading to an increased critical spacing for the U/U condition, consistent with the finding of Kooi et al. (1994). One proposed explanation for the greater errors with similar stimuli is increased confusion between the target and flankers (Strasburger, 2020, p. 28), causing a large number of flanker misreports (Strasburger et al., 1991; Strasburger, 2005; Strasburger & Malania, 2013). Flanker confusions would be impossible in the U/L condition used by Harrison and Bex (2014). Finally, although not tested here, grouping of the target and/or flankers may be modulated by target/flanker similarity, leading to configural effects that impact performance (Herzog et al., 2015).

Interestingly, even in the same visual field (right visual field) and when the same letter was used for both target and flankers, critical spacing differed depending on whether letter U or T was used. On average, the critical spacing was 1.33× larger with T stimuli than with U stimuli for the 67-ms condition and 1.25× larger for the 500-ms condition. In other words, the specific choice of letters for the target and flankers, even in the absence of a target-flanker dissimilarity, could affect the critical spacing, despite the report that critical spacing is invariant with the types of stimulus (letters and everyday objects: Wallace & Tjan, 2011; components of words and faces: Martelli et al., 2005). The difference in critical spacing for different letters is likely due to the different legibility of letters (Reich & Bedell, 2000), as well as the different cues or features used by subjects under crowded conditions.

Using letter Ts as both the target and flankers, we found that the critical spacing is larger in the lower visual field than in the right visual field for all subjects. Averaged across subjects, the critical spacing is approximately 1.47× and 1.44× larger in the lower than in the right visual field for the 67-ms and 500-ms condition, respectively. Previously, Toet and Levi (1992) and Chung (2013) showed that the crowding zones are almost always larger in the lower visual field than in the visual field along the horizontal meridian,^1^ by approximately a factor of 1.31×. This difference in size is not due to the radial-tangential anisotropy because it is observed along both the radial and tangential meridians (or the major and minor axes) of a crowding zone.

These results suggest that the small critical spacing reported by Harrison and Bex (2014) is likely the combined effects of the use of flankers that were different from the target, the choice of the letter U as the target, and their choice of the right visual field as the testing location. Our empirical study suggests that had they used the letter T for both the target and flankers and tested their subjects in the lower visual field, the results would be very comparable with those in the literature, similar to the T/T lower visual field (LVF) condition that we did, which were included in the curve-fitting in Figure 8. Note also that the stimulus manipulations (target-flanker similarity, choice of specific letter as target and flankers, and visual field location) affect critical spacing for both the 67-ms and 500-ms target/flanker duration, although the effects are smaller for the 500-ms duration. In other words, there is an interaction of each of these effects and duration on limiting the critical spacing, as confirmed by our statistical results (see above).

### Four flankers vs. two radial flankers

**Figure A.1.**
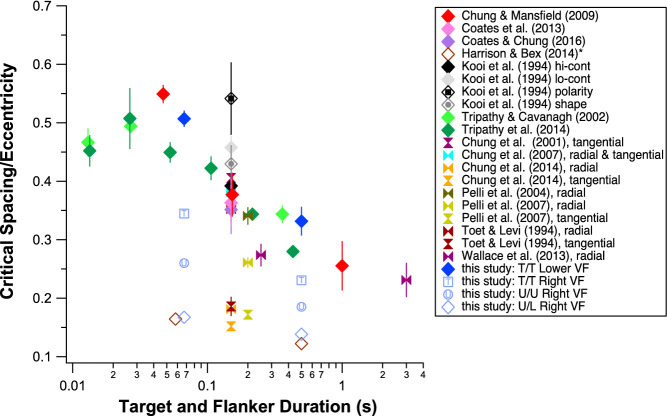
Ratio of critical spacing to eccentricity (Bouma fraction) is plotted as a function of target/flanker duration for studies that presented four flankers (diamond symbols, same as Figure 8), two radial flankers (bowtie symbols), or two tangential flankers (hourglass symbols). The empirical findings from this study, are also plotted (target U flanked by random letters and target U flanked by Us in the right visual field, target T flanked by Ts in the right and lower visual fields; see legend and text for details).

Another stimulus factor that can modulate the crowding effect is the number of flankers. Strasburger et al. (1991) found a difference in contrast thresholds when identifying numbers flanked by two flankers versus four flankers. Using Gabor stimuli as target and flankers, Põder and Wagemans (2007) showed that performance for identifying a target dropped from 55% correct with two flankers arranged radially with respect to the target and fixation to 38% correct with four flankers surrounding the target. In contrast, using letters as stimuli, Pelli et al. (2004) found no difference in the critical spacing whether a target letter was flanked by two radial flankers or four flankers. As mentioned earlier, in addition to studies that presented four flankers with the target, we have also reviewed studies that presented only two flankers, one on each side of the target along either the radial or the tangential direction with respect to the target and fixation. Appendix Figure A.1 plots all the Bouma fraction as a function of target/flanker duration for all the studies that we have reviewed. Diamond symbols refer to studies in which four flankers were used (as in Figure 8). Bowtie symbols represent studies or conditions in which two flankers were presented with the target along the radial direction, and hourglass symbols refer to the conditions in which two flankers were presented along the tangential direction. In some cases, authors measured the critical spacing for the radial and tangential flanker conditions separately within the same studies (see legend in Figure A.1).

As shown in Figure A.1, the bowtie symbols (representing data for the two-radial-flanker conditions) fall quite close to the cluster of data obtained using four flankers, but not for the hourglass symbols (two-tangential-flanker conditions). This is not surprising given that radial-tangential anisotropy is one of the signatures of crowding in normal peripheral vision (Levi, 2008; Nandy & Tjan, 2012) and that the critical spacing is generally 1.5 to 2.5× larger along the radial than the tangential direction (Chung, 2013; Nandy & Tjan, 2012; Pelli et al., 2007; Toet & Levi, 1992). The important question is, how does the critical spacing measured with only two radial flankers compare with that measured with four flankers? To address this question, we fit a linear function to the set of Bouma fractions with duration, as we did in Figure 8, but this time included the data obtained using two radial flankers. The slope of the fitted line is −0.17 ± 0.01, highly similar to that of the line fitted only to the four-flanker data (Figure 8), suggesting that the critical spacings are similar whether four flankers, one along each cardinal direction with respect to the target, or two flankers along the radial axis, were used. The important implication of this finding is that the radial flankers exhibit “winner-takes-all” behavior: They are not simply more important than the tangential flankers in limiting the size of the critical spacing, but they are the only flankers that matter, because adding the two tangential flankers does not change the size of the critical spacing.

Several of the studies included in Figure A.1 (Pelli et al., 2004, 2007; Wallace et al., 2013) that presented only two flankers used contrast threshold as the performance measurement, instead of proportion correct for identification of the target. Despite the differences between the two approaches, there does not seem to be any difference in the critical spacing measured using the two approaches.

## Conclusion

Since the pioneering work of Bouma (1970), there have been many investigations of the characteristics of the critical spacing, or how various stimulus factors impact the size and shape of the critical spacing. To unify the results from different psychophysical methods, we found that a general model of crowded identification matched diverse studies, allowing the extrapolation of the arbitrary landscape of flanked performance. In this article, we sought to search for any generalities that relate to the size of the critical spacing. Here, we are going to summarize these generalities in the context of an investigator who is faced with the task of deciding on the experimental conditions for a crowding study.

*Should I measure percent-correct performance or contrast threshold as the independent variable?* **** Critical spacings are comparable for these two measurements, and thus the choice would depend on the research question.

*If I measure acuity, should I vary the stimulus size or the target-flanker spacing? If it is the latter, should I use nominal or absolute spacing?* **** As we show in Figure 5, varying the stimulus size or target-flanker spacing is equivalent to sampling the size-spacing plot in different dimensions. As long as the stimulus size is chosen appropriately, the two methods should yield equivalent results. As for nominal or absolute spacing, they can be converted to one another easily, but the use of absolute spacing (center-to-center) may make it easier to relate to the concept of an integration field.

*Which psychophysical paradigm to use?* **** As illustrated in Figure 5, there is a parameter space describing crowded letter identification that can be sampled in a variety of different ways. Although different psychophysical procedures may lead to slightly different results (e.g., staircases may have bias vs. method of constant stimuli), in principle, the ability to identify a crowded stimulus of a given size and flanker spacing should have the same performance level, no matter how it is measured.

*Should I use the same stimulus for both target and flankers?* Critical spacing is larger when the same stimulus is used for both target and flankers (e.g., the same letter but with different orientations) and smaller when the flankers are different from the target (e.g., different letters). For example, we found that the critical spacing was 1.34 to 1.55× larger when a target letter U was flanked by four other letter Us than when it was flanked by four other letters.

*Choice of letters as target and flankers:* **** Even if the same letter is used for target and flankers, the choice of the specific letter as stimulus would affect the absolute size of the critical spacing. Admittedly, we have not tested all the letters, but the critical spacing seems to be similar for letter Ts and E, and is approximately 1.3× larger than for letter Us.

*Two versus four flankers:* **** As long as the two flankers are arranged radially with respect to the target and fixation, the critical spacing is similar whether two or four flankers are used the “winner-takes-all” behavior. Critical spacing is smaller if the two flankers are arranged tangentially with respect to the target and fixation (radial-tangential anisotropy).

*Contrast of target and flankers:* **** When the contrast of the target and flankers is reduced in yoked fashion, the critical spacing increases for contrasts below approximately 20%, reaching 1.5× high-contrast critical spacing at a contrast of 2.7%. In the fovea, however, the critical contrast is higher and the effect of contrast is more pronounced, likely because the larger target size needed for low-contrast identification overcomes the effect of the flankers.

*Duration of target and flankers:* **** Critical spacing is largest around 20 to 30 ms and reduces with increased (and decreased) duration. For every one log unit increase in duration, the Bouma fraction is reduced by 0.16, or the critical spacing is reduced by 0.16 * eccentricity.

*Visual field location:* Previous studies (Toet & Levi, 1992; Chung, 2013) and the empirical results in this article showed that at the same eccentricity, the critical spacing is approximately 1.3 to 1.5× larger in the lower visual field than in a field location along the horizontal meridian (right/left visual field).

*What threshold criteria should be used?* **** Many possible criteria have been used, which will change the critical spacing; stricter criteria—nearer to the asymptote of the crowding function—may yield significantly larger critical spacings.

*Should the results be plotted on a logarithmic or linear abscissa?* **** We are in favor of plotting psychometric functions of performance versus spacing on a logarithmic abscissa, since empirical results are consistent with a logarithmic effect (specifically, psychometric slopes are the same only when using the logarithm of flanker spacing), like many phenomena in visual science.

*What is the result of combining stimulus manipulations?* **** Following from the logarithmic spacing axes recommendation, stimulus combinations should have *multiplicative* effects on the critical spacing.

Besides potentially useful for designing an experiment, these generalities would facilitate comparisons of critical spacing across studies that used different experimental conditions. Additionally, any model of crowding or suggestions of the neural site of crowding would need to take these findings into consideration.

## Footnotes

1 Because some studies tested the nasal visual field, while others referred to the right or left visual field, here, we refer to all these visual field locations as “visual field along the horizontal meridian.”
